# East London Hospital for Children

**Published:** 1905-05-27

**Authors:** 


### EAST LONDON HOSPITAL FOR CHILDREN.

### THE HISTORY OF THE HOSPITAL.

The Duke of Portland presided at the festival dinner of the East London Hospital for Children, which was held at the Savoy Hotel on Monday, May 22nd. Amongst those present were the Earl of Arran, the Earl of Errol, the Earl of Albemarle, Viscount Falkland, Lord and Lady Castletown, the Marchioness Cassar de Sain, the Hon. Catherine Cary, Sir Harry and Lady Samuel, Sir Henry and Lady Vavasour, and Mr. Hugh Colin Smith.

The Chairman, in proposing the toast of "The East London Hospital for Children," briefly narrated the history of what he called this truly great and noble work of philanthropy. It was founded, he said, by a very good and heart-whole man, one Dr. Heckford, entirely at his own cost, in a warehouse at Ratcliff Cross, in January 1868. He, together with his noble wife, devoted the whole of his life and strength to its interests until his untimely death at the early age of 29 in 1871. A few days before that sad event, the site of the present hospital was purchased by the committee which had been formed to carry on and also to enlarge the scope of the work which Dr. Heckford had inaugurated. The advantage of the great and good work of the hospital was so apparent that it of course, as every good work would, quickly enlarged itself, and from its being the merely private and philanthropic work of one man, it had become an absolute necessity to the poor people in whose midst it was situated. Dr. Heckford, in 1868, inaugurated the work, with only one small ward containing 10 little iron bedsteads, there were now 3 wards for general, medical, and surgical cases, containing 97 beds and an isolation block of 10 beds, to which on an average 1,700 children of all ages up to 14 years were admitted annually. In the out-patient department too, some 35,000 individual cases were treated during the year. If for a moment they compared the 10 little beds inaugurated in 1868, and the numbers of patients he had just quoted his hearers would see the powerful necessity there was for the children's hospital at Shadwell. The Board of Management encouraged, as it had been, by liberal grants from King Edward's Fund, had erected two isolation wards of 6 beds each, to receive whooping-cough, the mortality from which in that district was appalling. There was required before next December, if the hospital were not to have to face a considerable deficit, £4,750, as two bad financial years in succession had rendered it necessary to sell part of the small invested capital to pay off a loan from the bankers. Besides that £4,750, it was necessary to raise £1,400 in order to secure a legacy of £3,000 which had been left on that condition and a grant of £500 from the Company of Drapers towards the cost of erecting these whooping-cough wards and isolation block.

Lord Castletown replied. He mentioned that in days gone by, when funds were running short, Charles Dickens had visited the hospital and wrote an article in which he so touched the feelings of those who were able to help that £1,000 was placed in Dr. Heckford's hands, and the closing of wards, which was then imminent, was prevented.

Replying for the "Board of Management and the Medical Staff," Col. Needham referred to the loss the hospital had sustained in the appointment of the late secretary to the position of clerk to St. Bartholomew's Hospital. For 11 years Mr. Hayes had devoted himself to the service of the hospital, and had recently left, to the great regret of the Board of Management, to take up one of the highest positions in the hospital world.

The amount collected as the result of the dinner was announced as being £966.

